# Factors Predicting Ventilator Dependence in Patients with Ventilator-Associated Pneumonia

**DOI:** 10.1100/2012/547241

**Published:** 2012-07-31

**Authors:** Chia-Cheng Tseng, Kuo-Tung Huang, Yung-Che Chen, Chin-Chou Wang, Shih-Feng Liu, Mei-Lien Tu, Yu-Hsiu Chung, Wen-Feng Fang, Meng-Chih Lin

**Affiliations:** ^1^Division of Pulmonary and Critical Care Medicine, Department of Internal Medicine, Kaohsiung Chang Gung Memorial Hospital, Chang Gung University College of Medicine, Kaohsiung 833, Taiwan; ^2^Department of Respiratory Care, Chang Gung University of Science and Technology, Chiayi 813, Taiwan; ^3^Department of Respiratory Therapy, Kaohsiung Chang Gung Memorial Hospital, Chang Gung University College of Medicine, Kaohsiung 833, Taiwan; ^4^Division of Pulmonary and Critical Care Medicine, Xiamen Chang Gung Hospital, Xiamen, China

## Abstract

*Objectives*. To determine risk factors associated with ventilator dependence in patients with ventilator-associated pneumonia (VAP). *Study Design*. A retrospective study was conducted at Chang Gung Memorial Hospital, Kaohsiung, from January 1, 2007 to January 31, 2008. *Methods*. This study evaluated 163 adult patients (aged ≥18 years). Eligibility was evaluated according to the criterion for VAP, Sequential Organ Failure Assessment (SOFA) score, Acute Physiological Assessment and Chronic Health Evaluation II (APACHE II) score. Oxygenation index, underlying comorbidities, septic shock status, previous tracheostomy status, and factors related to pneumonia were collected for analysis. *Results*. Of the 163 VAP patients in the study, 90 patients survived, yielding a mortality rate of 44.8%. Among the 90 surviving patients, only 36 (40%) had been weaned off ventilators at the time of discharge. Multivariate logistic regression analysis was used to identify underlying factors such as congestive cardiac failure (*P* = 0.009), initial high oxygenation index value (*P* = 0.04), increased SOFA scores (*P* = 0.01), and increased APACHE II scores (*P* = 0.02) as independent predictors of ventilator dependence. Results from the Kaplan-Meier method indicate that initial therapy with antibiotics could increase the ventilator weaning rate (log Rank test, *P* < 0.001). *Conclusions*. Preexisting cardiopulmonary function, high APACHE II and SOFA scores, and high oxygenation index were the strongest predictors of ventilator dependence. Initial empiric antibiotic treatment can improve ventilator weaning rates at the time of discharge.

## 1. Introduction

Ventilator-associated pneumonia (VAP) is a hospital-acquired (nosocomial) condition developing more than 48 hours after the introduction of mechanical ventilation. The estimated incidence is 9–27%, with a mortality rate of 25–50% [1–5]. VAP is one of the most common nosocomial infections, complicating the treatment of patients in intensive care units (ICUs). VAP remains a major cause of morbidity and mortality among critically ill patients and incurs excessive medical expenses for institutions [6]. Early diagnosis and treatment is important because appropriate management of VAP can be an issue of life and death [7, 8]. Recently, an algorithm was proposed, with which to evaluate the benefits of early tracheostomy for patients susceptible to prolonged ventilator dependence [9]. However, identifying patients who will require long-term mechanical ventilation remains a challenge.

Many patients who survive VAP encounter problems while being weaned from the ventilator, thereby developing chronic ventilator dependence. Nearly all patients with prolonged dependence on mechanical ventilation suffer from a profound impairment in physical function and/or cognitive status at the time of discharge, and most require institutional care [10]. Chronic ventilator dependence is a serious medical problem and an extremely uncomfortable condition for patients, with considerable social and financial implications [11, 12]. For example, at-home use of ventilators requires ongoing supervision, and patients often misinterpret clinical deterioration for equipment problems [13]. 

Because no predictive factors have previously been identified for ventilator weaning failure among VAP patients, this study sought to identify these independent risk factors. It has been demonstrated that appropriate initial empiric antibiotic therapy can increase the survival rate of patients with VAP [14]; therefore, this study endeavored to determine whether such therapy could shorten the duration of ventilator dependence. We hypothesize that appropriate initial empiric use of antibiotics may be helpful in preventing ventilator dependence. Underlying comorbidities and the severity of disease may have a profound influence on clinical outcomes.

## 2. Patients and Methods

### 2.1. Setting and Study Design

This retrospective study was conducted at Kaohsiung Chang Gung Memorial Hospital, a 2400-bed tertiary teaching hospital in southern Taiwan. The study was conducted in 4 medical ICUs and 3 surgical ICUs including 70 beds from January 1, 2007 to January 31, 2008 for adult intubated patients (aged ≥18 years) presenting with nosocomial pneumonia pathologically diagnosed and confirmed as well as confirmed microbiologically by sputum cultures. Patients known to have tuberculosis or severe immunosuppression, such as those with human immunodeficiency virus (HIV) or solid-organ or bone marrow transplantation, were excluded from the study. The enrolled patients were divided into two groups, ventilator independent and ventilator dependent, according to their status at the time of discharge from the hospital.

 The study was approved by the Institutional Review Board of Chang Gung Memorial Hospital, and the requirements for patient consent were waived.

### 2.2. Definitions

Pneumonia was defined according to modified criteria proposed by the United States Center for Disease Control and Prevention [15]. This definition requires that two of the following criteria be satisfied: fever (increase in body temperature of ≥1°C or body temperature >38.3°C), leukocytosis (25% increase and leukocyte count ≥ 10,000 mm^3^) or leukopenia (25% decrease and leukocyte count ≤ 5,000 mm^3^), and purulent tracheal secretions (>25 neutrophils per high-power field). It also requires that one of the following be satisfied: new and persistent infiltrates appearing on chest radiograph, the same microorganisms isolated from pleural fluid and tracheal secretions, radiographic cavitation, histological proof of pneumonia, or positive cultures from bronchoalveolar lavage (≥1 × 10^4^ colony-forming units/mL). To ensure the accuracy of the VAP diagnosis, a modified Clinical Pulmonary Infection Score (CPIS) was calculated for every patient, and patients with CPIS below and equal to six were excluded. VAP was defined as the occurrence of pneumonia following 48–72 hours of mechanical ventilation [16, 17]. To avoid confounding the initial diagnosis of pneumonia and subsequent VAP, patients with pneumonia resulting in respiratory failure had to have undergone successful treatment for the initial episode of pneumonia. Ventilator dependence was defined as the failure to wean the patient from the ventilator while hospitalized in the intensive care unit or respiratory care center, in conjunction with continued use of a ventilator according to hospital discharge status. Polymicrobial infection was defined as more than two pneumonia-causing microorganisms cultured from the same respiratory sample. Acute respiratory distress syndrome (ARDS) status prior to the occurrence of VAP was defined as arterial oxygen tension (Pa02)/fractional inspired oxygen (Fi02) < 200.

### 2.3. Initial Empiric Antibiotic Therapy

This clinical strategy emphasizes prompt empiric therapy for all suspected cases of VAP due to the high mortality rates, if the initiation of appropriate antibiotic therapy is delayed [7, 18]. Thus, this study complied with the 2005 Infectious Disease Society of America/American Thoracic Society (IDSA/ATS) guidelines for hospital-acquired pneumonia [5] and the selection of initial antibiotic therapy was based on risk factors for specific pathogens, modified by a knowledge of local patterns in antibiotic resistance and the prevalence of specific organisms. Therapy was modified based on clinical response at days 2 and 3 and the results of semiquantitative cultures of secretions from the lower respiratory tract. Patients without risk factors for multiple drug resistant (MDR) strains received limited-spectrum antibiotics, similar to those used for community-acquired pneumonia, as suggested by the guidelines. However, in our ICU, most patients with VAP received broad-spectrum antibiotics due to the risk of MDR pathogens. Broad-spectrum therapies should cover *Pseudomonas aeruginosa* (the most common pathogen in an ICU setting) and include third- or fourth- generation cephalosporin, a carbapenem and a beta-lactam/beta lactamase inhibitor, in addition to a quinolone or aminoglycoside. In suspected cases of methicillin-resistant *Staphylococcus aureus*, initial coverage would include vancomycin or linezolid. This approach requires no specialized microbiologic methods, and avoids the problem associated with a failure to treat some infected individuals. After stabilizing the patients, we focused on de-escalating treatment according to the results of semiquantitative cultures to minimize drug resistance and reduce costs [19]. Antibiotic appropriateness was determined according to an evaluation of the initial response to empiric antibiotic treatment for underlying pneumonia. After treatment with antibiotics for seven days, the clinical status of treatment was reevaluated and classified as “appropriate” if the fever subsided, sputum production had decreased, pneumonia infiltration regression was observed on the chest radiograph, and laboratory data had improved. If clinical condition at days 2 and 3 after antibiotic treatment become worse and antibiotic therapy should be modified, the treatment was classified as “inappropriate.” If clinical status could not be determined as “appropriate” or “inappropriate,” it was designated as “indeterminate.”

### 2.4. Ventilator Weaning Protocol

Ventilator weaning profile is protocolized in our ICU [20–22]. Patients are ready to be weaned from ventilators when the underlying cause of acute respiratory failure indicates improvement or resolution, adequate gas exchange is observed as indicated by a Pa02 above 60 mm Hg while breathing with a FI02 of 0.40 or less with a positive end-expiratory pressure of 5 cm H20 or less, a core temperature below 38°C, and no further need for vasoactive or sedative agents. In addition, the attending physician had to agree that the patient was in stable condition and ready to be weaned from the ventilator. Patients underwent a trial of spontaneous breathing for 120 minutes when the rapid shallow breathing index (respiratory rate/tidal volume) was <105 breaths/min/L and maximal inspiratory pressure was below −20 cm H20. Patients failing to meet these criteria when tested were reevaluated on a daily basis. Respiratory therapists terminated the trial if the patient had any of the following signs: a respiratory rate exceeding 35 breaths/min, oxygen saturation below 90%, heart rate above 140 beats/min, or a sustained increase or decrease in the heart rate of more than 20%, systolic blood pressure above 200 mm Hg or below 80 mm Hg, and agitation, diaphoresis, or anxiety. If a patient had any of these signs of poor tolerance at any time during the trial, mechanical ventilation was reinstituted. Patients demonstrating no signs of poor tolerance at the end of the trial were immediately extubated and provided supplemental oxygen by a face mask.

### 2.5. Data Collection

Clinical data retrieved from medical records included age, gender, initial admitting diagnosis, Sequential Organ Failure Assessment (SOFA) score during the occurrence of VAP, Acute Physiological Assessment and Chronic Health Evaluation II (APACHE II) score during the occurrence of VAP, underlying comorbidities, the reasons behind respiratory failure, septic shock status, acute respiratory distress syndrome (ARDS) status prior to the occurrence of VAP, and tracheostomy status before and after occurrence of VAP. Comorbidity was quantified using the Charlson comorbidity index, as previously described [23]. Pneumonia-related characteristics such as ventilation days before the occurrence of VAP, cultured organisms, polymicrobial infection status, CPIS score, procedure of bronchoalveolar lavage (BAL), appropriateness of initial empiric antibiotics, initial FI02 use, initial Pa02 values, initial mean airway pressure (MAP), initial oxygenation index (OI, OI = Fi02 × MAP/Pa02) during occurrence of VAP, and initial vital signs were also recorded to compare differences between ventilator-dependent and ventilator-independent subjects. All variables were evaluated as possible predictors of ventilator dependence. The aim of this study was to elucidate the independent factors used to predict ventilator dependence.

### 2.6. Statistical Analysis

Categorical variables were analyzed using the chi-squared test or Fisher's exact test where appropriate, and continuous variables were compared using Student's *t*-test or the Mann-Whitney* U* test. Multivariate logistic regression analysis was performed to identify risk factors for ventilator dependence. All variables considered as risk factors with a *P* value <0.10 in univariate analysis were entered into the multivariate model. If individual variables associated with ventilator dependence had a *P* value < 0.05 in the multivariable model, a backward elimination procedure was used to identify the final independent risk factors. Receiver operating characteristic (ROC) curves were plotted and the area under the curve was compared with general severity scores measured in this study. The cutoff value of general severity scores for predicting ventilator dependence among VAP patients were analyzed according to ROC curves. The proportion of patients undergoing ventilation was estimated by means of Kaplan-Meier analysis comparing subjects receiving appropriate and inappropriate initial empiric antibiotics. We also used the Kaplan-Meier method to illustrate the relevance among four stratified Pa02/Fi02 classes (stratified as values >400, 300 ~ 400, 200 ~ 300, and <200) and the duration of ventilation. Differences in ventilator weaning were calculated according to logrank statistics.

Results are presented as absolute numbers (percentage) or mean ± standard deviation (SD). Adjusted odds ratios (AORs) and 95% confidence intervals (CIs) were reported for logistic regression analysis. A two-tailed *P* value of <0.05 was considered significant. All statistical analysis was performed using the SPSS 14.0 software package (SPSS Inc., Chicago, IL, USA).

## 3. Results

### 3.1. Patient Characteristics

A total of 798 patients diagnosed with pneumonia were admitted into the hospital within a 13-month period (January 1, 2007 to January 31, 2008). We found that 163 patients met the criteria for diagnosis with VAP. Among the 163 patients, 73 patients died and 90 patients survived while hospitalized, for a mortality rate of 44.8%. Among the 90 surviving patients, 54 were ventilator dependent at the time of discharge from the hospital, and 36 patients were ventilator free. The ventilator weaning rate among VAP patients was only 40%. The mean APACHE II score (SD) in patients with ventilator dependence is 25.76 (3.75) and 23.25 (4.72) in patients with ventilator independence (Table 2). The mean APACHE II score (SD) in VAP survivors is 24.66 (4.32) and in 26.52 (3.72) VAP nonsurvivors.

The initial diagnoses of VAP survivors at the time of admission are presented in Table 1. It was found that 79 patients had been admitted with variable initial diagnoses and 11 patients had been admitted for surgical reasons. Baseline characteristics of patients are presented in Table 2. Between the ventilator-dependent and ventilator-independent groups, no statistical differences were observed in age (*P* = 0.91), sex (*P* = 0.55), Charlson comorbidity index values (*P* = 0.91), status of septic shock (*P* = 0.67), underlying comorbidities such as liver cirrhosis (*P* = 0.34), end-stage renal disease (*P* = 0.93), neoplastic disease (*P* = 0.36), diabetes mellitus (*P* = 0.65), or previous cerebrovascular accident status (*P* = 0.86). However, higher SOFA scores (*P* < 0.001), higher APACHE II scores (*P* = 0.006), pneumonia or chronic obstructive pulmonary disease causing acute respiratory failure (*P* < 0.001), ARDS status prior to the occurrence of VAP (*P* < 0.001) and underlying comorbidities such as congestive heart failure (*P* < 0.001) and chronic respiratory disease (*P* = 0.001) were frequently noted in ventilator-dependent patients. Overall tracheostomy status (*P* = 0.20) and tracheostomy following the occurrence of VAP (*P* = 0.29) did not contribute statistically to high ventilator weaning rates. Conversely, tracheostomy performed prior to the occurrence of VAP (*P* = 0.004) had a greater chance of leading to ventilator dependence. This implies that early tracheostomies may be performed in patients with poor cardiopulmonary function when ventilator dependence is anticipated (Table 2).

### 3.2. VAP Characteristics

VAP-related characteristics are shown in Table 3. We found that ventilation days prior to the occurrence of VAP (*P* = 0.29), varieties of pneumonia-causing organism (*P. aeruginosa *(*P* = 0.34), *A. baumanii *(*P* = 0.69), *S. matophilia *(*P* = 0.18), *K. pneumonia *(*P* = 0.46), *S. aureus *(*P* = 0.92)), polymicrobial infection status (*P* = 0.42), initial Pa02 values (*P* = 0.88), CPIS score (*P* = 0.45), BAL procedure (*P* = 0.08), and initial vital signs such as temperature (*P* = 0.31), heart rate (*P* = 0.56), and mean blood pressure (*P* = 0.25) were not positively associated with ventilator dependence among VAP patients. Nevertheless, we also observed that appropriate initial treatment with empiric antibiotics (*P* < 0.001), low initial Fi02 use (*P* = 0.003), low initial MAP (*P* = 0.002), low initial OI value (*P* < 0.001), and high initial respiratory rate (*P* = 0.02) increased the likelihood of ventilator independence in VAP patients.

### 3.3. Predictors of Ventilator Dependence

Using univariate analysis of factors capable of predicting ventilator dependence, it was found that ventilator-dependent survivors had statistically higher APACHE II scores (AOR 1.15, 95% confidence interval (CI) 1.04−1.28, *P* = 0.008) and SOFA scores (AOR 1.79, 95% CI 1.38−2.31, *P* < 0.001) than ventilator-free survivors. Ventilator-dependent survivors also had a statistically greater likelihood of having underlying congestive heart failure (AOR 6.25, 95% CI 2.24−17.47, *P* < 0.001), chronic respiratory disease (AOR 5.76, 95% CI 1.95−17.04, *P* = 0.002), higher initial Fi02 use (AOR 1.09, 95% CI 1.03−1.15, *P* = 0.005), higher initial MAP (AOR1.12, 95% CI 0.85−1.21, *P* = 0.004), higher OI values (AOR 1.28, 95% CI 1.14−1.44, *P* < 0.001), respiratory failure with pneumonia (AOR 0.55, 95% CI 0.42 ~ 0.79, *P* < 0.001), previous ADRS status prior to the occurrence of VAP (AOR 7.75, 95% CI 2.62 ~ 22.97, *P* < 0.001), lower initial respiratory rate (AOR 0.93, 95% CI 0.86–0.99, *P* = 0.03), and previous tracheostomy (AOR 5.97, 95% CI 1.62−22.07, *P* = 0.007) than ventilator-free survivors. Initial treatment with appropriate empiric antibiotics (AOR 2.87, 95% CI 1.46−5.64, *P* = 0.002) was also predictive of ventilator independence. Multivariate logistic regression analysis was used to identify independent factors and underlying congestive heart failure (AOR 15.58, 95% CI 1.97 ~ 123.54, *P* = 0.009), initial high OI value (AOR 1.44, 95 % CI 1.01 ~ 2.06, *P* = 0.04), increased SOFA scores (AOR 1.67, 95% CI 1.12−2.50, *P* = 0.01), and increased APACHE II scores (AOR 1.35, 95% CI 1.04−1.74, *P* = 0.02) as independent predictors of ventilator dependence (Table 4).

### 3.4. Receiver Operating Characteristic Curves

**Figure 1:**
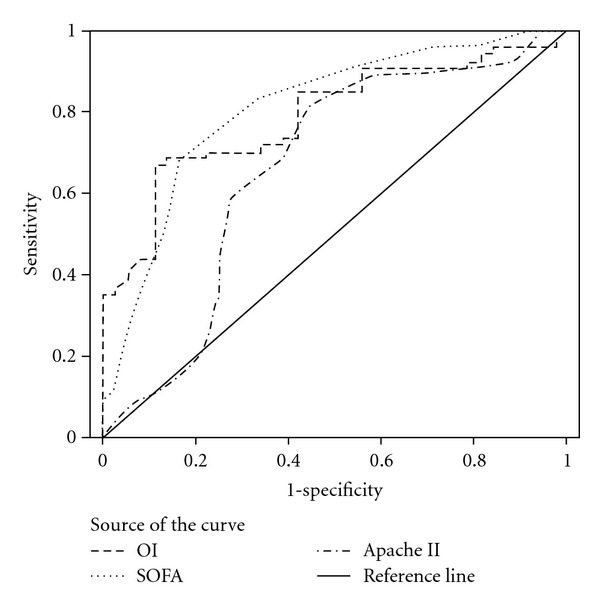
ROC curve analysis for predictability of ventilator dependence between SOFA score, APACHE IIscore and OI.

ROC curves were plotted to identify cutoff values that would best determine ventilator dependence (Figure 1). The optimal cutoff values for SOFA score, OI, and APACHE II score were 8.5, 12.01, and 23.5, respectively. These values yielded a sensitivity and specificity of 83% and 67%, for the SOFA score, 68% and 89% for OI, and 82% and 57% for APACHE II, respectively. The area under the ROC curve indicated higher sensitivity and specificity for the SOFA score than OI and APACHE II score for determining ventilator dependence (0.81 versus 0.79 and 0.66, *P* < 0.0001).  Table 5 shows the comparison of cutoff values, sensitivity, specificity, AUC, and *P*-value for these three physiologic scores.

### 3.5. Appropriateness of Initial Empiric Antibiotics and Ventilator Weaning

**Figure 2:**
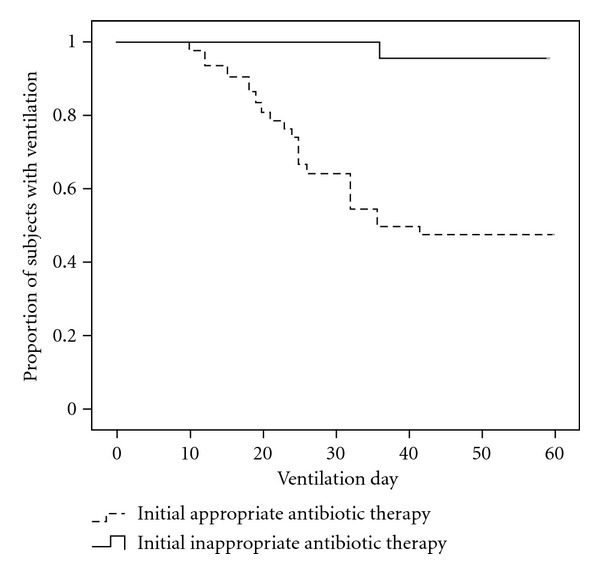
Kaplan-Meier curve showing the proportion of patients with ventilation over time according to initial appropriate or inappropriate antibiotic therapy.

To determine the relationship between the appropriateness of initial empiric antibiotics and success in ventilator weaning, we used the Kaplan-Meier plot to indicate relevance (Figure 2). We found that appropriate initial empiric antibiotic therapy was strongly associated with ventilator weaning, and the curves differed statistically based on the logrank test (*P* < 0.001).

### 3.6. Relevance of Pa02/Fi02 Stratified Values and Ventilation Duration

**Figure 3:**
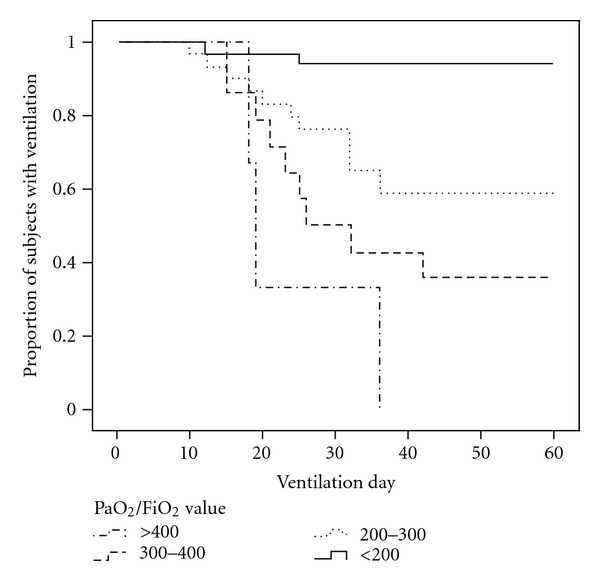
Kaplan-Meier curve showing the proportion of patients with ventilation over time according to stratified PaO2/FiO2 values.

To illustrate the correlation between impaired oxygenation status during the occurrence of VAP and prolonged mechanical ventilation, we stratified Pa02/Fi02 value in four classes (>400, 300 ~ 400, 200 ~ 300, and <200) using the Kaplan-Meier method. In a Kaplan-Meier plot (Figure 3), we can see the values of the four classes as distinct from one another. The class with Pa02/Fi02 value > 400 had a shorter ventilation duration, the class with Pa02/Fi02 value < 200 had the longest ventilation duration, and the others two classes (value between 300 ~ 400, 200 ~ 300) fell between. This suggests that impaired oxygenation status during the occurrence of VAP influences ventilation duration and the curves were determined to be statistically different based on the logrank test (*P* < 0.001).

## 4. Discussion

This retrospective analysis yielded three main findings. First, patients with VAP may have high rates of ventilator dependence. Walkey et al. suggested that patients with VAP were more prone to ventilator dependence than with patients without VAP [24]. Second, clinical variables such as underlying chronic cardiopulmonary disease, previous ADRS, high initial Fi02 and MAP support, high initial OI, high APACHE II and SOFA scores during the occurrence of VAP, COPD with acute exacerbation, a low initial respiratory rate, history of tracheostomy before the occurrence of VAP, and inappropriate initial use of antibiotics may have a negative influence on ventilator weaning rates. Patients with respiratory failure due to pneumonia including community acquired pneumonia, healthcare-associated pneumonia, and hospital acquired pneumonia supervening with ventilator associated pneumonia have a higher likelihood of developing ventilator dependence, despite the successful initial treatment of pneumonia. In addition, we determined that previously impaired cardiac function and high OI, APACHE II, and SOFA scores during the occurrence of VAP were independent factors in predicting ventilator dependence. Third, the appropriateness of initial empiric antibiotics was an important factor related to ventilator weaning among patients with VAP. Fourth, Pa02/Fi02 values stratified as four groups as >400, 300 ~ 400, 200 ~ 300, and <200 were highly correlated with ventilation duration.

Patients can become dependent on ventilator support for a variety of reasons. Often, hospitalized elderly patients who are nutritionally depleted develop pneumonia leading to respiratory failure [25, 26]. When patients have impaired underlying lung function and suffer from infections such as pneumonia, they fail to oxygenate the blood properly, leading to respiratory failure and the need for mechanical ventilation. When reversible conditions such as infection and malnutrition are properly and completely addressed, patients can be successfully weaned from their dependence on ventilators.

In this study, we found that underlying congestive heart failure and chronic respiratory disease may depress the ventilator-weaning rate in patients with VAP, and these underlying cardiopulmonary diseases may lead to impaired pulmonary function. If patients with cardiopulmonary diseases acquire VAP while being hospitalized, they are likely to suffer from ventilator dependence. Previous studies have suggested that cardiac surgery can lead to decreased pulmonary function (as measured by spirometry) and impaired pulmonary mechanics—even in the absence of diaphragmatic dysfunction—for several months following surgery [27–30], and this may lead to the need for prolonged mechanical ventilation. COPD results from inflammation and/or alterations in repair mechanisms, the “spill-over” of inflammatory mediators into the circulation, may result in important systemic manifestations of the disease, such as wasting of skeletal muscle and cachexia [31–33]. Furthermore, skeletal muscle can be wasted by sepsis syndrome [34]. This acquired weakness results in prolonged mechanical ventilation and difficulty in weaning.

Using several scoring systems, intensive care physicians are able to accurately and reliably measure the severity of illness in the ICU. Most scoring systems focus on mortality as the main outcome measure. Disease severity as indicated by the APACHE II and SOFA scores also influences ventilator dependence in VAP patients in this study. Siempos and Vardakas suggested that disease severity and SOFA on the day of diagnosis with VAP could be independent predictors for mortality in meta-analysis [35]. Gursel and Demirtas suggested that APACHE II scores at the time of diagnosis with VAP could be useful in predicting mortality in the pulmonary ICU population developing VAP [36]. We also found that mortality and ventilator dependence were both related to poor APACHE II and SOFA scores. If patients receive an APACHE II score of >23.5 and a SOFA score of >8.5, this would imply profoundly compromised lung function and may increase the difficulty associated with ventilator weaning.

Oxygenation failure at any point during the course of acute hypoxemic respiratory failure has an impact both on the duration of mechanical ventilation and survival. This is best reflected by OI [37]. Rapid deterioration of OI in intubated ventilated patients is associated with high mortality rates; a diminished improvement of OI appears to increase the risk of death in the course of acute respiratory failure. This emphasizes the importance of OI in monitoring oxygenation status in ventilated patients. Gajic et al. also suggested that age, OI, and cardiovascular failure three days after intubation are predictors of death or prolonged mechanical ventilation, and may inform decisions regarding specific interventions such as tracheostomy, particularly in terms of the design of clinical trials [38]. In this study, we also determined that poor cardiopulmonary function and initial OI could be independent factors for predicting ventilator dependence, particularly among VAP patients with OI value over 12.01.

Mechanical ventilation leads to high morbidity, mortality, and financial cost. Because prolonged or delayed liberation from ventilators can cause harm, safe, expeditious weaning is highly desirable [39–41]. Aggressive therapy to reduce the ventilatory workload should be pursued. In patients with nosocomial infections, appropriate initial antibiotic therapy is associated with increased survival rates, reduced hospital stays, and lower healthcare costs [42, 43]. Broad-spectrum antibiotics are an optimal initial choice for nosocomial pneumonia and severe sepsis [44]. In this study, we also found that the appropriateness of initial empiric antibiotic therapy for patients with VAP may be helpful in weaning patients from mechanical ventilation. Timely administration of antibiotics is associated with decreased mortality as well as reduced impairment to the inflammatory response [42]. If delaying the initiation of antibiotic therapy precludes prompt abatement of systemic inflammation, this would arouse intense inflammatory mediator responses. Pro-inflammatory mediators can be activated with consequent uncontrolled activation of the immune system causing lung damage, leading to acute lung injury or ARDS that would further damage underlying pulmonary function and eliminate any chance of ventilator weaning.

Mechanical ventilation may cause substantial morbidities such as VAP, further destroying pulmonary function. If patients under mechanical ventilation cannot be liberated in a timely manner, prolonged ventilation contributes to an increase in clinical morbidity running in a vicious cycle. This makes weaning an important clinical issue for patients and clinicians. 

The important discovery of the present study is that initial appropriate empiric treatment with antibiotics shortens the duration with which ventilation must be maintained by addressing the underlying impairment to cardiopulmonary function. Disease of high severity also leads to ventilator dependence. A number of limitations should be mentioned when interpreting the results of this study. First, the retrospective design of the study may have passed over factors influencing the outcome. It is our hope that the design would generate hypotheses for future prospective controlled studies to confirm our final points. Second, bronchoalveolar lavage was not performed in every patient in this retrospective study. However, sputum cultures from endotracheal aspirate were used as a diagnostic method in patients suspected of carrying pulmonary infection. Third, ventilator settings could be one important factor that could contribute to ventilator dependence, and protective ventilation strategy may be helpful in ARDS patient. However, ventilation strategy was not described in this study. Indeed, we could not record ventilation setting in a retrospective manner because ventilator setting for VAP-ARDS patient is different individually by attending personnel. We also addressed this as a limitation.

## 5. Conclusion

VAP is associated with high mortality and can result in ventilator dependence, particularly among patients with underlying cardiopulmonary disease. Our findings suggest that elevated APACHE II and SOFA scores and higher OI at the time of VAP onset are independent factors capable of predicting ventilator dependence. Only appropriate initial empiric antibiotic therapy is capable of remedying this critical situation, resulting in successful liberation from ventilators.

## Figures and Tables

**Table 1:** Initial admitting diagnosis in VAP^a^ survivors.

	Initial admitting diagnosis	Case No^a^
	Community-acquired pneumonia	4
	Health-care associated pneumonia	10
	COPD^a^ with acute exacerbation	12
	Congestive heart failure with acute pulmonary edema	11
	Acute myocardial infarction	5
	Acute stroke	5
	Upper gastrointestinal bleeding	6
Medical diagnosis	Uremia	8
	Terminal malignancy	3
	Urinary tract infection	6
	Skin infection	3
	CNS^a^ infection	3
	L-spine^a^ compression fracture	1
	Decompensated liver cirrhosis with massive ascites	1
	Organophosphate intoxication	1

	Total	79

	Head injury with intracerebral hemorrhage	5
Surgical diagnosis	Hollow organ perforation	3
	C-spine^a^ injury	2
	Benign prostate hyperplasia	1

	Total	11

^a^CNS: Central nervous system, COPD: chronic obstructive pulmonary disease, C-spine: cervical spine, L-spine: lumbar spine, No: number.

**Table 2:** Baseline VAP patients characteristics^a^.

Characteristics	Ventilator independence	Ventilator dependence	*P* value
	*N* = 36	*N* = 54	
Age	66.08 ± 15.69^d^	66.46 ± 14.66^d^	0.91

Sex			0.55
Male, number (%)	21 (58)^d^	28 (52)^d^	
Female, number (%)	15 (42)^d^	26 (48)^d^	

SOFA^b^ score	7.61 ± 2.42^d^	10.41 ± 2.02^d^	<0.001

Apache II score	23.25 ± 4.72^d^	25.76 ± 3.75^d^	0.006

Reason of respiratory failure			<0.001
Pneumonia	5 (14)^d^	18 (33)^d^	
COPD^b^ with AE^b^	6 (17)^d^	19 (35)^d^	
Cardiogenic	6 (17)^d^	12 (22)^d^	
GI^b^ bleeding	2 (6)^d^	0 (0)^d^	
Terminal malignancy	4 (11)^d^	2 (4)^d^	
Other infections	13 (36)^d^	3 (6)^d^	

Charlson comorbidity Index^c^	2.36 ± 1.29^d^	2.39 ± 1.79^d^	0.91

ARDS^b^ status	5	30	<0.001

Comorbidities			
Chronic lung disease	5 (14)^d^	26 (48)^d^	0.001
CHF^b^	6 (17)^d^	30 (56)^d^	<0.001
Liver cirrhosis	2 (6)^d^	1 (2)^d^	0.34
End-stage renal disease	5 (14)^d^	7 (13)^d^	0.93
Neoplastic disease	3 (8)^d^	8 (15)^d^	0.36
Diabete mellitus	13 (36)^d^	17 (31)^d^	0.65
Old CVA^b^	16 (44)^d^	23 (43)^d^	0.86

Septic shock	8 (22)^d^	10 (19)^d^	0.67

Tracheostomy before VAP^b^	3 (8)^d^	19 (35)^d^	0.004

Tracheostomy after VAP^b^	10 (28)^d^	9 (17)^d^	0.29

Overall tracheostomy	13 (36)^d^	28 (52)^d^	0.20

^a^ Continuous variables were analyzed by Student's *t*-test or Mann-Whitney *U* test, and categorical data by chi-square test.

^b^ AE: acute exacerbation, APACHE: Acute Physiological Assessment and Chronic Health Evaluation, ARDS: acute respiratory distress syndrome, CHF: congestive heart failure, COPD: chronic obstructive pulmonary disease, CVA: cerebrovascular accident, GI: gastrointestinal, SOFA: Sequential Organ Failure Assessment, VAP: ventilator associated pneumonia.

^c^ Disease was defined as in the Charlson comorbidity index.

^d^ Variables are expressed as mean (standard deviation) and categorical data are expressed as number (percentage).

**Table 3:** Ventilator-associated pneumonia related characteristics^a^.

Characteristics	Ventilator independence	Ventilator dependence	*P* value
	*N* = 36	*N* = 54	
Ventilation day before VAP^b^	9.58 ± 4.66^c^	10.76 ± 5.44^c^	0.29

Organism, number (%)			
* P. aeruginosa*	13 (36)^c^	25 (46)^c^	0.34
* A. baumannii*	8 (22)^c^	14 (26)^c^	0.69
* S. maltophilia*	4 (11)^c^	12 (22)^c^	0.18
* K. pneumoniae*	4 (11)^c^	9 (17)^c^	0.46
* S. aureus *	9 (25)^c^	13 (24)^c^	0.92

Polymicrobial infection, number (%)	11 (31)^c^	21 (39)^c^	0.42

CPIS^b^	8.64	8.83	0.45

BAL^b^, number (%)	10 (28)^c^	25 (46)^c^	0.08

Appropriateness of initial empiric antibiotic, number (%)			<0.001
Appropriate	30 (83)^c^	20 (37)^c^	
Inappropriate	1 (3)^c^	22 (41)^c^	
Indeterminate	5 (14)^c^	12 (22)^c^	

Initial Fi02^b^ (%)	41.31 ± 8.24^c^	48.43 ± 12.09^c^	0.003

Initial MAP^b^	18.78 ± 5.45^c^	23.01 ± 6.71^c^	0.002

Initial OI^b^	7.81 ± 3.55^c^	14.19 ± 7.34^c^	<0.001

Initial Pa02^b^	105.36 ± 43.52^c^	103.89 ± 45.46^c^	0.88

Initial vital sign			
Temperature	37.74 ± 1.35^c^	38.34 ± 3.86^c^	0.31
Respiratory rate	23.04 ± 8.21^c^	19.76 ± 4.99^c^	0.02
Heart rate	98.86 ± 28.14^c^	95.91 ± 19.43^c^	0.56
Mean blood pressure	85.31 ± 16.56^c^	89.70 ± 5.52^c^	0.25

^a^Continuous variables were analyzed by Student's *t*-test or Mann-Whitney *U* test, and categorical data by chi-square test.

^b^BAL: bronchoalveolar lavage, CPIS: Clinical Pulmonary Infection Score; FI02: fraction inspired oxygen, MAP: mean airway pressure, OI: oxygenation index, Pa02: arterial oxygen tension, VAP: ventilator associated pneumonia.

^c^Variables are expressed as mean (standard deviation) and categorical data are expressed as number (percentage).

**Table 4:** Predictors of ventilator dependence in patients with ventilator-associated pneumonia by multivariate logistic regression analysis.

Predictors	Odd ratio (95 % CI)	*P* values
Congestive heart failure	15.58 (1.97–123.54)	0.009
Initial oxygenation index	1.44 (1.01–2.06)	0.04
SOFA^a^ score	1.67 (1.12–2.50)	0.01
APACHE^a^ II score	1.35 (1.04–1.74)	0.02

^a^APACHE: Acute Physiological Assessment and Chronic Health Evaluation, SOFA: Sequential Organ Failure Assessment.

**Table 5:** Comparison of cutoff value, sensitivity, specificity, AUC^a^, and *P*-value between physiological severity scores.

Factors	Cutoff value	Sensitivity	Specificity	AUC	*P*-value
SOFA^a^ score	8.5	0.83	0.67	0.81	<0.001
Oxygenation index	12.01	0.68	0.89	0.79	<0.001
APACHE^a^ II score	23.5	0.82	0.57	0.66	0.009

^a^ APACHE: Acute Physiological Assessment and Chronic Health Evaluation, AUC: area under curve, SOFA: Sequential Organ Failure Assessment.

## Abbreviations

AORs: Adjusted odd ratios
APACHE: Acute Physiological Assessment and Chronic Health Evaluation
ARDS: Acute respiratory distress syndrome
BAL: Bronchoalveolar lavage
CI: Confidence interval
COPD: Chronic obstructive pulmonary disease
CPIS: Clinical Pulmonary Infection Score
FI02: Fraction inspired oxygen
HIV: Human immunodeficiency virus
ICU: Intensive care unit
IDSA/ATS: Infectious Diseases Society of America/American Thoracic Society
MAP: Mean airway pressure
MDR: Multiple drug resistant
OI: Oxygenation index
Pa02: Arterial oxygen tension
ROC: Receiver operating characteristic
SD: Standard deviation
SOFA: Sequential Organ Failure Assessment
VAP: Ventilator-associated pneumonia.
